# Identification of novel cell glycolysis related gene signature predicting survival in patients with endometrial cancer

**DOI:** 10.1186/s12935-019-1001-0

**Published:** 2019-11-14

**Authors:** Zi-Hao Wang, Yun-Zheng Zhang, Yu-Shan Wang, Xiao-Xin Ma

**Affiliations:** Department of Obstetrics and Gynecology, Key Laboratory of Obstetrics and Gynecology of Higher Education of Liaoning Province, Shengjing Hospital of China Medical University, 39 Huaxiang Road, Shenyang, 110021 People’s Republic of China

**Keywords:** Endometrial cancer, Glycolysis, Prognostic, mRNA, Survival

## Abstract

**Background:**

Endometrial cancer (EC) is one of the three major gynecological malignancies. Numerous biomarkers that may be associated with survival and prognosis have been identified through database mining in previous studies. However, the predictive ability of single-gene biomarkers is not sufficiently specific. Genetic signatures may be an improved option for prediction. This study aimed to explore data from The Cancer Genome Atlas (TCGA) to identify a new genetic signature for predicting the prognosis of EC.

**Methods:**

mRNA expression profiling was performed in a group of patients with EC (n = 548) from TCGA. Gene set enrichment analysis was performed to identify gene sets that were significantly different between EC tissues and normal tissues. Cox proportional hazards regression models were used to identify genes significantly associated with overall survival. Quantitative real-time-PCR was used to verify the reliability of the expression of selected mRNAs. Subsequent multivariate Cox regression analysis was used to establish a prognostic risk parameter formula. Kaplan–Meier survival estimates and the log‐rank test were used to validate the significance of risk parameters for prognosis prediction.

**Result:**

Nine genes associated with glycolysis (*CLDN9*, *B4GALT1*, *GMPPB*, *B4GALT4*, *AK4*, *CHST6*, *PC*, *GPC1*, and *SRD5A3*) were found to be significantly related to overall survival. The results of mRNA expression analysis by PCR were consistent with those of bioinformatics analysis. Based on the nine-gene signature, the 548 patients with EC were divided into high/low-risk subgroups. The prognostic ability of the nine-gene signature was not affected by other factors.

**Conclusion:**

A nine-gene signature associated with cellular glycolysis for predicting the survival of patients with EC was developed. The findings provide insight into the mechanisms of cellular glycolysis and identification of patients with poor prognosis in EC.

## Background

Endometrial cancer (EC) is one of the three major gynecological malignancies and fifth most common cancer among women (4.8% of female cancer cases) in the United States [1]. It is expected that 61,880 new cases will be diagnosed in 2019 (7% of all female cancer cases) and 12,160 deaths (4% of all female cancer deaths). In the past ten years, with the irregular use of hormones and changes in people’s living environment and lifestyle, the prevalence and mortality of endometrial cancer in China and abroad have been increasing annually [2]. Although most patients are diagnosed early, approximately 28% of patients are diagnosed with advanced disease. However, patients with the same degree of progression can show different prognoses and treatment responses. Therefore, effective EC biomarkers must be discovered for assessing prognosis and identifying potentially patients at a high risk of EC.

Numerous biomarkers for EC have been identified, such as the SIX1 and HER-2 genes [3, 4]. With advancements in high-throughput sequencing, researchers have established various patient genome databases to enable a more systematic understanding of genomic changes. Through database mining, we identified thousands of biomarkers that may be associated with the prognosis of patients with tumors [5, 6]. However, the predictive ability of single-gene biomarkers remains insufficient. Studies have shown that the evaluation of genetic traits, which involve multiple genes, may improve prognosis prediction [7, 8]. Multigenic prognostic features from primary tumor biopsy can guide more specific treatment strategies. Recent studies have explored the effects of multiple-gene signature on EC for assessing prognosis and identifying potentially patients at a high risk of EC [9, 10].

In this study, genes were selected by performing gene set enrichment analysis (GSEA). To identify biomarkers, differential analysis typically involves comparison of the expression differences between groups and focuses on genes whose expression levels are significantly regulated. However, this method can easily exclude genes that do not show obvious expression differences but may provide important biological information or exhibit biological significance. As an emerging computational method, GSEA does not require a clear differential gene threshold or extensive experience to test the overall expression of several genes. It reveals general trends in the data. Therefore, this approach improves the statistical analysis between biological expression and biological significance [11].

Accordingly, in the present study, we aimed to explore data from The Cancer Genome Atlas (TCGA) to identify a new genetic signature for predicting the prognosis of EC. To this end, we used mRNA expression data from TCGA to map the marker genome of 548 patients with EC. We identified 119 mRNAs significantly related to glycolysis and developed a nine-gene risk profile for effectively predicting patient outcomes. Interestingly, the risk factors associated with glycolysis can be used assess prognosis of high-risk patients independently. A novel cell glycolysis-related gene signature was identified and validated.

## Methods

### Clinical information and mRNA expression data set of patients

We extracted clinical data and the mRNA expression profiles of patients with endometrial cancer from TCGA (https://cancergenome.nih.gov/) [12]. The study included clinical information from 548 patients and enrolled matching age, stage, grade, radiation therapy, residual tumor, histological type, diabetes, new tumor events, and hypertension (Table 1).

**Table 1:** Clinical pathological parameters of patients with Endometrioid cancer in this study

Clinical pathological parameters	N	%	Dead number
Age			
≥ 66	236	43.2	47
< 66	310	56.8	40
Neoplasm cancer status			
With tumor	79	15.5	48
Tumor free	431	8.5	35
Residual tumor			
R0	376	94.5	47
R1	22	5.5	5
Stage			
I	341	62.2	29
II–IV	207	37.8	58
New event			
No	485	88.5	54
Yes	63	11.5	33
Grade			
G1	99	18.4	2
G2	122	22.7	14
G3	316	58.6	65
Histological type			
Endometrioid adenocarcinoma	411	75	46
Serous adenocarcinoma/mixed	137	25	41
Radiation therapy			
No	517	94.3	84
Yes	31	5.7	3
Diabetes			
No	533	97.3	86
Yes	15	2.7	1
Hypertension			
No	517	94.3	85
Yes	31	5.7	2

### Gene set enrichment analysis

We performed GSEA (http://www.broadinstitute.org/gsea/index.jsp) to determine if the identified gene sets were significantly different between the EC and normal groups. Next, we analyzed the expression levels of 24,991 mRNAs in EC samples and in adjacent noncancerous tissues. Finally, we determined functions for subsequent analysis by using normalized p values (p < 0.05).

### Data processing and risk-parameter calculation

Log2 transformation was used to normalize each mRNA from among the expression profiles. Univariate Cox regression analysis was used to identify genes associated with overall survival (OS), which were then subjected to multivariable Cox regression to confirm the genes related to prognosis and obtain the coefficients. The selected mRNAs were then divided into the risky (hazard ratio, HR > 1) type and protective (0 < HR < 1) type. By linearly combining the expression values of filtered genes weighted by their coefficients, we constructed a risk-parameter formula as follows: Risk parameter = ∑ (βn × expression of gene n). Using the median risk parameter as a cut-off, the 548 patients were divided into high‐risk and low‐risk subgroups.

### Specimens and patients of quantitative real-time (qRT)-PCR

A total of 20 EC tissues and 20 normal endometrial tissues were obtained from patients at the Department of Gynecology and Obstetrics, Shengjing Hospital of China Medical University, China. Normal tissues were obtained from patients who underwent hysterectomy for endometrial-irrelevant diseases. All patients provided informed consent, and this study was approved by the Ethics Committee of Shengjing Hospital of China Medical University. Histological diagnosis and grade were assessed by experienced pathologists in accordance with the FIGO 2009. No patient was administered systemic treatment preoperatively.

### RNA extraction and qRT-PCR

Total RNA was extracted from tissues using TRIzol reagent (Vazyme, Nanjing, China). PrimeScript RT-polymerase (Vazyme) was used to reverse-transcribe cDNAs corresponding to the mRNAs of interest. qRT-PCR was performed using SYBR-Green Premix (Vazyme) with specific PCR primers (Sangon Biotech Co., Ltd, Shanghai, China). Glyceraldehyde-3-phosphate dehydrogenase was used as an internal control. The 2^−ΔΔCt^ method was used to calculate fold-changes. Primer sequences are listed in Additional file 1: Table S1.

### Statistical analysis

We used Kaplan–Meier survival curves and the log‐rank method to estimate the significance of the risk parameter. We performed multivariate Cox analysis and data stratification analysis to test whether the risk parameter was independent of the clinical features, including age, grade, stage, new event, residual tumor, and neoplasm cancer status, which were used as covariates. A p < 0.05 was considered as statistically significant. Statistical analyses were performed using GraphPad Prism7 software (GraphPad, Inc., La Jolla, CA, USA) and SPSS 20.0 software (SPSS, Inc., Chicago, IL, USA).

## Results

### Initial screening of genes by GSEA

**Fig. 1:**
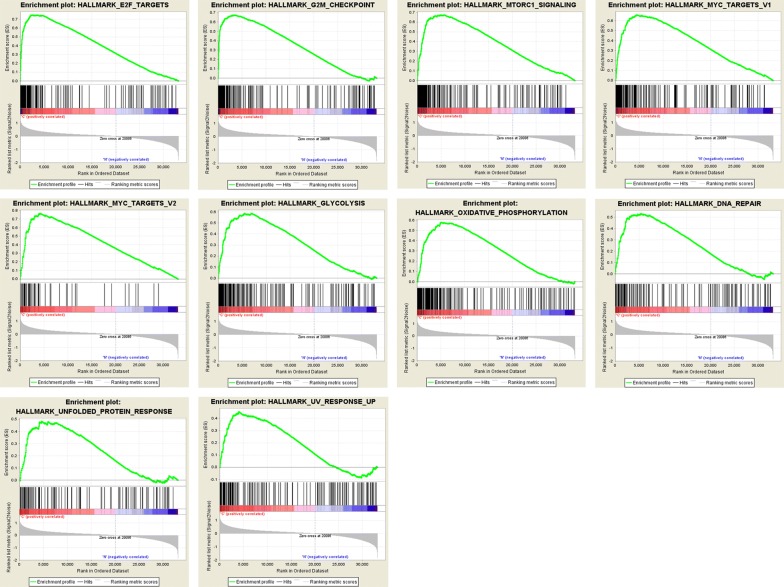
Enrichment plots of nine gene sets which had significant difference between noncancerous tissues and EC tissues by performing GSEA

We obtained the clinical features of 548 patients with EC, along with an expression data set for 24,991 mRNAs from TCGA database. We performed GSEA to determine if the identified gene sets were significantly different between EC tissues and endometrium tissues. We validated 26 gene sets that were upregulated in EC. Ten gene sets, G2 M checkpoints, MYC targets V1, glycolysis, MYC targets V2, MTORC1 signaling, oxidative phosphorylation, DNA repair, unfolded protein response, E2F targets, and UV response, were significantly enriched (Table 2, Fig. 1). We then filtered the top‐ranking function, glycolysis (p = 0.000), among 119 genes for subsequent analysis.

**Table 2:** Gene sets enriched in Endometrial cancer (548 samples)

GS follow link to MSigDB	SIZE	ES	NOM *p*‐value	Rank at MAX
E2F targets	199	0.749	0	2428
G2M checkpoint	198	0.674	0	3343
MTORC1 signaling	197	0.673	0	4792
MYC targets V1	199	0.657	0	4404
MYC targets V2	58	0.768	0	4140
Glycolysis	197	0.587	0	6922
Oxidative phosphorylation	199	0.578	0	5082
DNA repair	142	0.531	0	5276
Unfolded protein response	109	0.483	0	4590
UV response up	155	0.455	0	4007

### Identification of survival-associating glycolysis‐related mRNAs

First, we performed univariate Cox regression analysis of 119 genes for preliminary screening and obtained 21 genes (p < 0.05). Next, multivariate Cox regression analysis was performed to further examine the association between the 21 mRNA expression profiles and patient survival, and the stepwise elimination method was used to identify the most significant mRNAs combinations. Nine mRNAs (*CLDN9*, *B4GALT1*, *GMPPB*, *B4GALT4*, *AK4*, *CHST6*, *PC*, *GPC1*, and *SRD5A3*) were verified, as shown in Table 3, and six of the nine genes (*CLDN9*, *B4GALT1*, *GMPPB*, *AK4*, *PC*, and *SRD5A3*) were validated as independent prognostic markers of EC. The filtered mRNAs were divided into the risky type (*CLDN9*, *AK4*, *PC*, *GPC1*, and *SRD5A3*), with HR > 1 associated with poorer survival and the protective type (*B4GALT1*, *GMPPB*, *B4GALT4*, and *CHST6*), with HR < 1 associated with better survival (Table 3).

**Table 3:** The detailed information of nine prognostic mRNAs significantly associated with overall survival in patients with endometrial cancer

mRNA	Ensemble ID	Location	Β (Cox)	HR	p
CLDN9	ENSG00000213937	chr 16: 3,012,923–3,014,505	0.1059	1.1117	0.0258
B4GALT1	ENSG00000086062	chr 9: 33,104,082–33,167,356	− 0.2504	0.7785	0.0203
GMPPB	ENSG00000173540	chr 3: 49,716,844–49,723,951	− 0.4346	0.6475	0.0134
B4GALT4	ENSG00000121578	chr 3: 119,211,732–119,240,946	− 0.3041	0.7378	0.0839
AK4	ENSG00000162433	chr 1: 65,147,549–65,232,145	0.3181	1.3746	0.0015
CHST6	ENSG00000183196	chr 16: 75,472,052–75,495,445	− 0.1191	0.8878	0.0665
PC	ENSG00000173599	chr 11: 66,848,417–66,958,439	0.328	1.3882	0.0233
GPC1	ENSG00000063660	chr 2: 240,435,663–240,468,076	0.2056	1.2282	0.1022
SRD5A3	ENSG00000128039	chr 4: 55,346,242–55,373,100	0.2345	1.2643	0.05

**Fig. 2:**
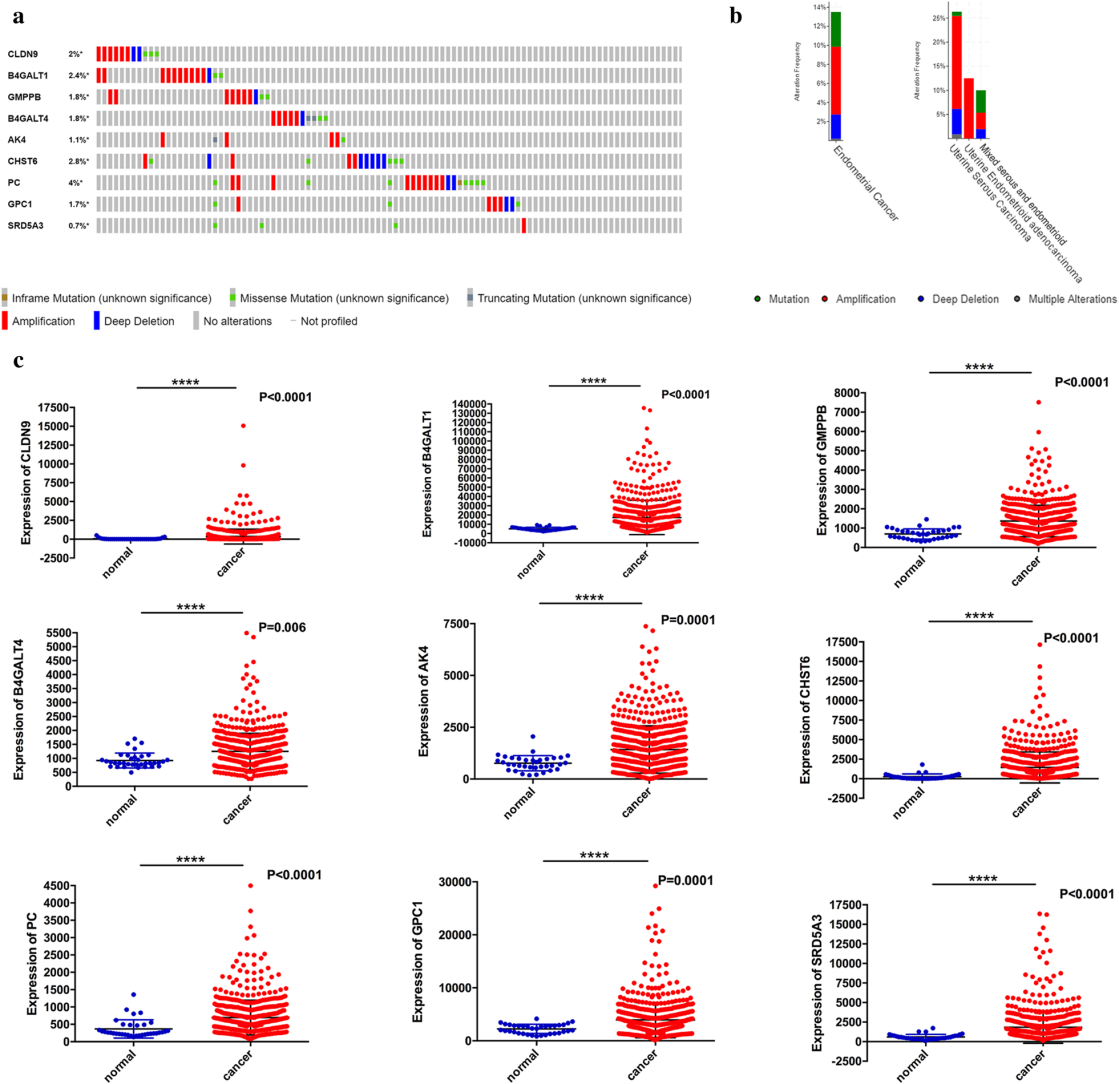
Identification of mRNAs related to patients’ survival. **a** Selected genes’ alteration in 548 clinical samples. **b** Selected genes’ specific alteration in different pathological types of EC. **c** Different expression of nine selected genes

We then assessed the alterations in nine filtered genes by analyzing 548 EC samples in the cBioPortal database (http://cbioportal.org) [13]. The results showed that the queried genes were altered in 98 (18.3%) of the sequenced cases. The PC gene included ten amplification samples, 2 deep deletion samples, 7 missense mutations samples, and one sample with an in-frame mutation. The CLDN9 gene was altered in 2% of cases, showing various changes. The CHST6 gene was altered in 2.8% of cases, and the B4GALT1 and B4GALT4 genes were altered in 2.4% and 1.8% of cases, respectively (Fig. 2a).

The specific alterations in the selected genes were significant in specific cancer types. In EC, 3.88% of changes were mutations, 7.17% were amplifications, 2.67% were deep deletions, and 0.16% were multiple alterations. In endometrioid adenocarcinoma, the only alteration was amplification (12.76% of patients). In mixed serous and endometrioid cases, the most eminent alteration was mutation (Fig. 2b).

Comparison of the expression differences of 9 genes between adjacent normal tissues and EC tissues was also performed. We found that the expression levels of the nine genes were significantly up- or downregulated in EC tissues (Fig. 2c).

### Validation of TCGA expression results using qRT-PCR

**Fig. 3:**
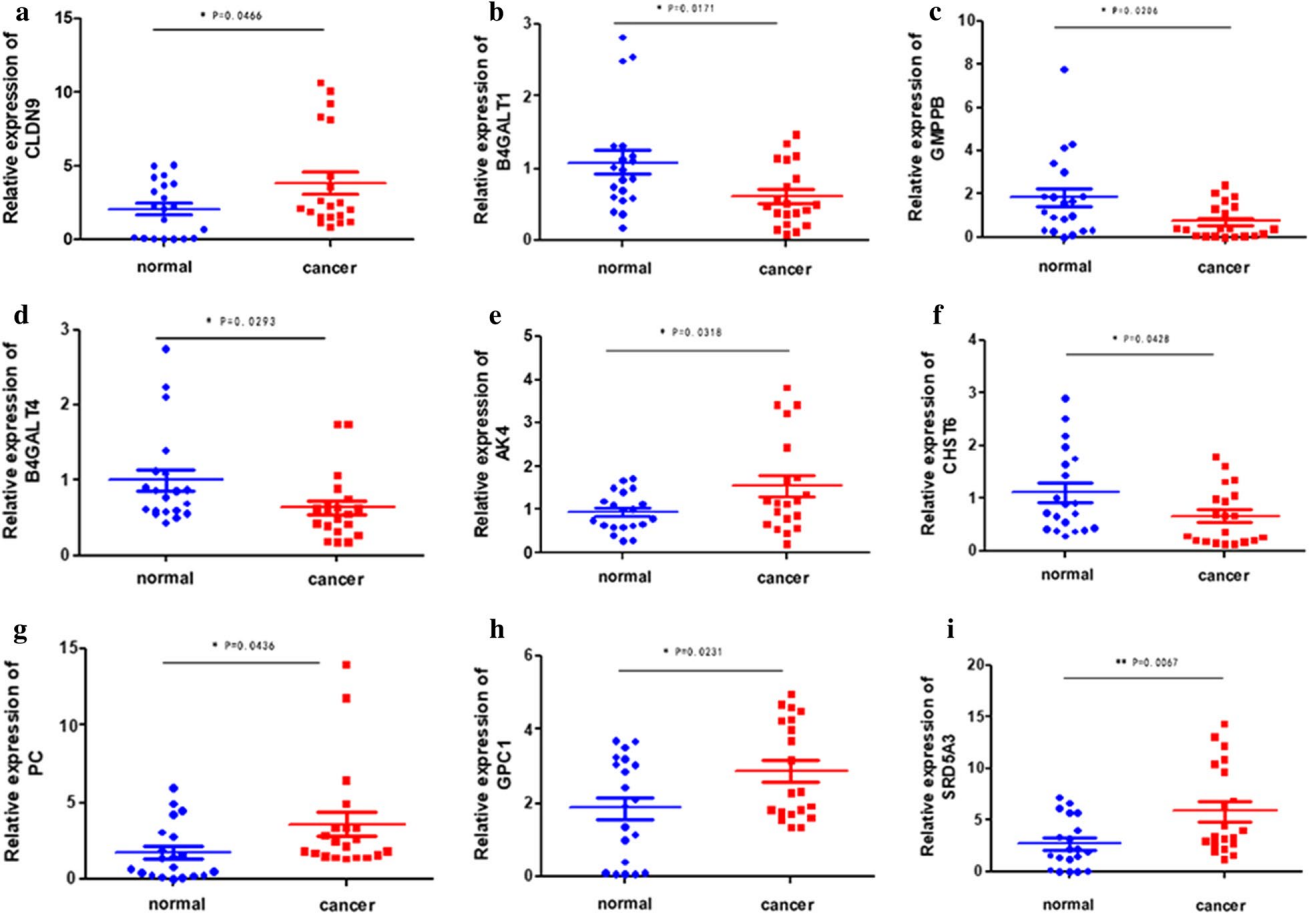
Expression of nine mRNAs in endometrial cancer tissues and normal tissues. **a** CLDN9, **b** B4GALT1, **c** GMPPB, **d** B4GALT4, **e** AK4, **f** CHST6, **g** PC, **h** GPC1, **i** SRD5A3

We examined the expression of 9 mRNAs by qRT-PCR in 20 EC tissues and 20 normal endometrial tissues. We applied the unpaired *t* test to assess the differences between the two groups. The results showed that *CLDN9*, *AK4*, *PC*, *GPC1*, and *SRD5A3* were upregulated in EC tissues compared to in normal endometrium tissues, whereas *B4GALT1*, *GMPPB*, *B4GALT4*, and *CHST6* were downregulated in tumor tissues (Fig. 3). The mRNA expression results of qRT-PCR validation in 20 patients with EC were consistent with the bioinformatics results, which showed that the bioinformatics analysis was precise and gave significant results.

### Construction of a nine‐mRNA signature to predict patient outcomes

**Fig. 4:**
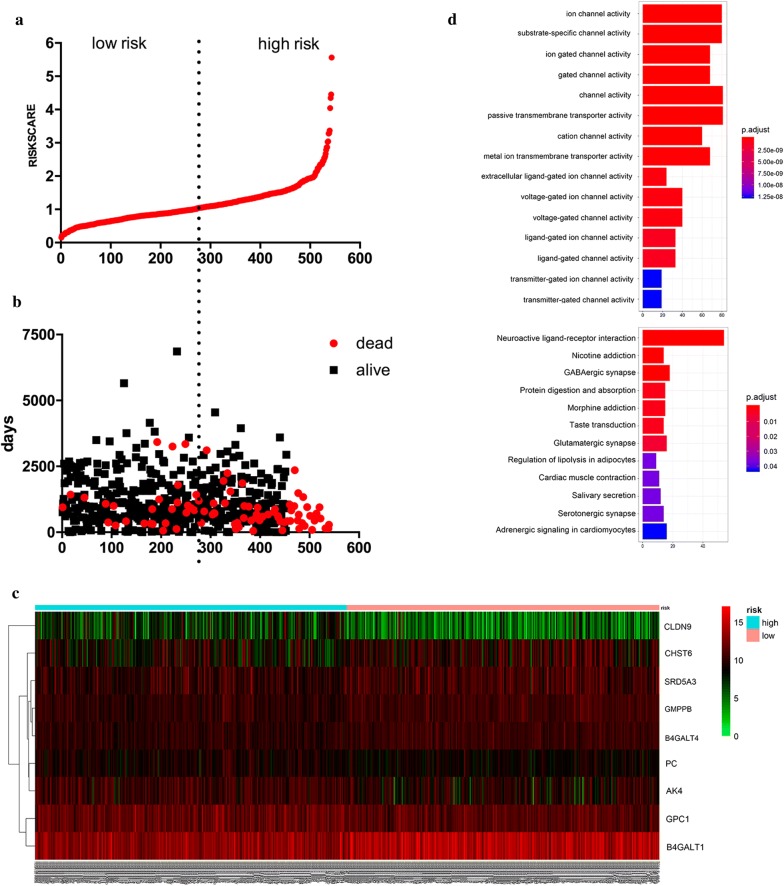
The nine‐mRNA signature associated with risk parameter predicts OS in patients with endometrial cancer. **a** mRNA risk parameter distribution in each patient. **b** Survival days of EC patients in ascending order of risk parameters. **c** A heatmap of nine genes’ expression profile. **d** GO analysis and KEGG analysis of nine differentially expressed mRNAs

By linearly combining the expression values of selected genes weighted by their coefficients derived from multivariate Cox regression analysis, we established the following prognostic risk-parameter formula. Risk parameter = 0.1059 × expression of *CLDN9* + 0.3181 × expression of *AK4* + 0.328 × expression of *PC* + 0.2056 × expression of *GPC1* + 0.2345 × expression of *SRD5A3* − 0.2504 × expression of *B4GALT1 *− 0.3041 × expression of *B4GALT4* − 0.4346 × expression of *GMPPB* − 0.1191 × expression of *CHST6*. We calculated parameters and assigned one risk parameter to each patient. We then ranked the patients in ascending order by the parameter and divided the patients into high-risk and low-risk subgroups using the median (Fig. 4a). The survival time of each patient is shown in Fig. 4b. Patients in the high‐risk parameter group showed poorer survival, whereas patients with the low‐risk parameter had lower mortality rates. Additionally, a heatmap displayed the expression profiles of nine mRNAs (Fig. 4c). Compared to the low-risk group, the expression level of risky-type mRNA (*CLDN9*, *AK4*, *PC*, *GPC1*, and *SRD5A3*) was higher in the high-risk group. In contrast, the expression level of protective-type mRNA (*CLDN9*, *AK4*, *PC*, *GPC1*, and *SRD5A3*) in the high-risk group was lower than that in the low-risk group. The expression levels of the nine genes in the high and low risk groups are shown in Additional file 2: Figure S1.

We performed Gene Ontology (GO) and Kyoto Encyclopedia of Genes and Genomes (KEGG) analyses of differentially expressed mRNAs, including KEGG pathway enrichment analysis and GO functional annotation analysis of the 9 differentially expressed genes in the high- and low-risk groups (Fig. 4d). KEGG pathway enrichment analysis showed that the differentially expressed genes were involved in multiple signaling pathways and enriched in neuroactive ligand-receptor interaction, protein digestion and absorption, regulation of lipolysis in adipocytes, and another 12 pathways. The two groups of differentially expressed genes were annotated with GO functions and analyzed for their significant functions. The results revealed 15 GO items in the significantly enriched molecular function category, including ion channel activity, substance-specific channel activity, and passive transmembrane transporter activity, among which passive transmembrane transporter activity was the most significantly enriched GO term.

### Risk parameter derived from nine-mRNA signature is an independent prognostic indicator

We compared the prognostic value of risk parameters with clinical pathology parameters by univariate and multivariate analyses (Table 1). Samples with well-established clinical data were selected. The median age of the 546 patients with EC was 66 years. Among the 548 patients, 63 (11.5%) had a new event during the follow‐up, 459 (75%) had endometrioid adenocarcinoma, and 31 (5.7%) suffered from hypertension. Among the 398 patients, 22 (5.5%) had residual tumors, and 99 (18.4%) had grade 1 tumors. Among 537 patients, 99(18.4%)had grade 1 tumors, 122 (22.7%) had grade 2 tumors, and the remaining 316 (58.6%) had grade 3 tumors. Furthermore, among the 548 patients with EC, 341 (62.2%) patients had stage I disease, and the remaining 207 (37.8) patients had stage II–IV disease. From the data set above, we identified the risk parameter, stage, grade, neoplasm cancer status, and new tumor event as independent prognostic indicators, as these factors showed significant differences in both univariate and multivariate analyses (Table 4). Notably, the risk parameters showed significant prognostic values with p < 0.05 (HR = 1.783).

**Table 4:** Univariable and multivariable analyses for each clinical feature

Clinical feature	Number	Univariate analysis			Multivariate analysis		
		HR	95%CI of HR	P value	HR	95%CI of HR	P value
Risk parameter (High-risk/Low-risk)	274/274	3.529	2.186–5.699	**< **0.001	2.482	1.832–4.258	0.038
Age (≥ 66/< 66)	236/310	1.817	1.180–2.798	0.007	1.482	0.820–2.678	0.192
Stage (I/II–IV)	341/207	3.577	2.288–5.594	**< **0.001	1.658	1.010–2.721	0.046
Grade (G1/G2–3)	99/438	12.811	3.148–52.128	**< **0.001	7.826	1.052–58.205	0.044
Residual tumor (yes/no)	22/376	2.884	1.778–4.678	**< **0.001	0.745	0.402–1.380	0.349
New tumor event (yes/no)	63/485	4.931	3.189–7.625	**< **0.001	2.773	1.604–4.798	**< **0.001
Neoplasm cancer status (with tumor/tumor free)	79/431	6.404	4.140–9.908	**< **0.001	3.509	1.895–6.498	**< **0.001
Histological type (endometrioid adenocarcinoma/others)	411/137	1.854	1.186–2.896	0.007	0.708	0.406–1.237	0.225

### Verification of nine‐mRNA signature for prognosis prediction by K–M survival estimates

**Fig. 5:**
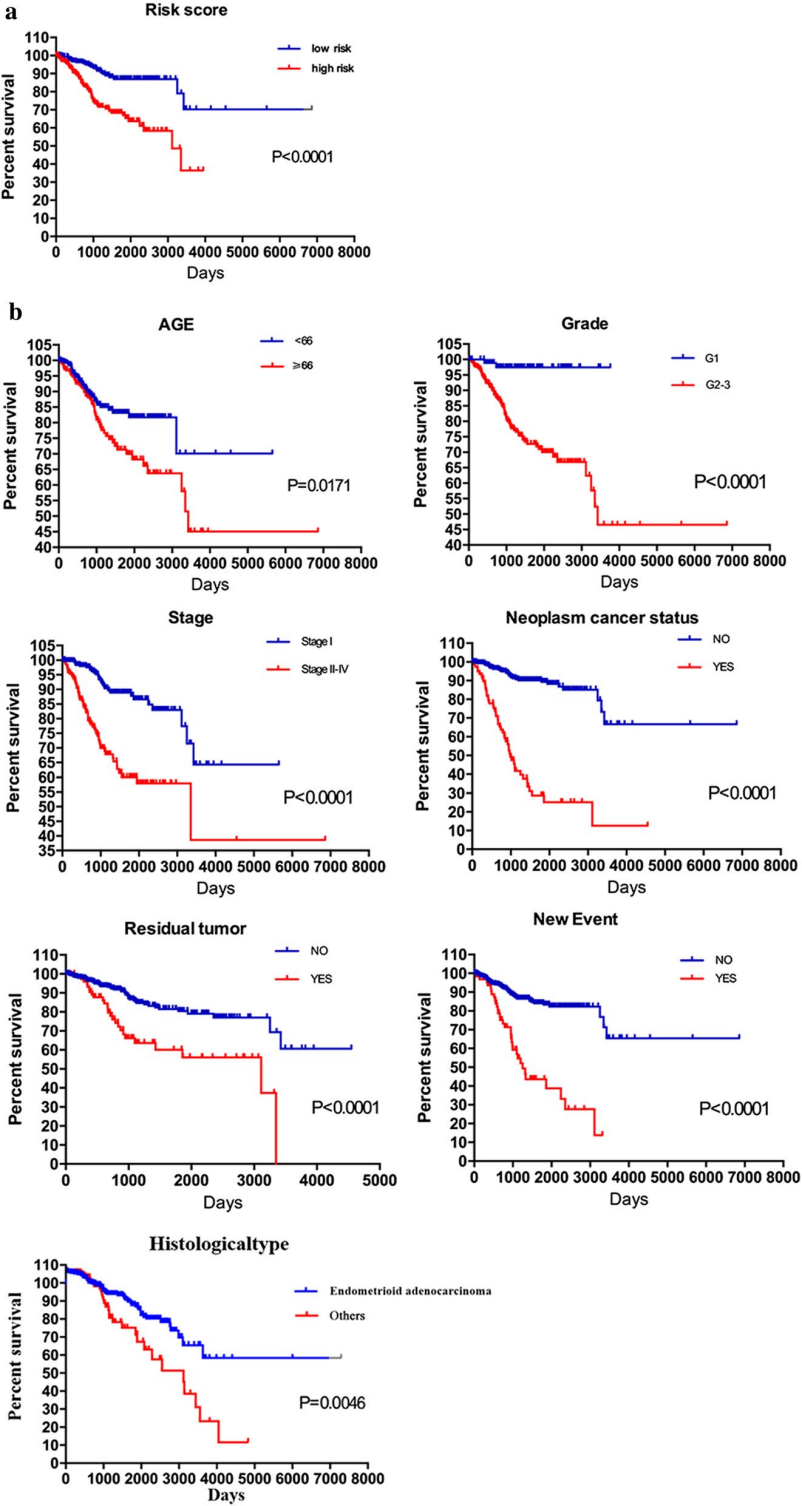
Kaplan–Meier survival analysis for EC patients in TCGA data set. **a** K–M survival curve for EC patients with high/low risk. **b** Clinical features including age, grade, stage, neoplasm cancer status, residual tumor and new event predict patients survival. **c** histological type predict patients survival

K–M survival estimates and the log‐rank test revealed that patients in the high-risk group had a poor prognosis (Fig. 5a). Univariate Cox regression analysis of OS revealed several clinicopathological parameters which were predictive of EC survival, including age, grade, stage, history of cancer, residual tumor, tumor recurrence (new events), and histological type. We then used the Kaplan–Meier survival estimates to validate the above conclusions, which showed consistent results, Patients older than 66 years, with tumor grade G2–3, disease stage II–IV, histological type of serous adenocarcinoma or mixed serous/endometrioid, and suffering from tumor recurrence (new events) and residual tumors were associated with poor prognosis (Fig. 5b, c). These results further confirmed the reliability of the analysis.

**Fig. 6:**
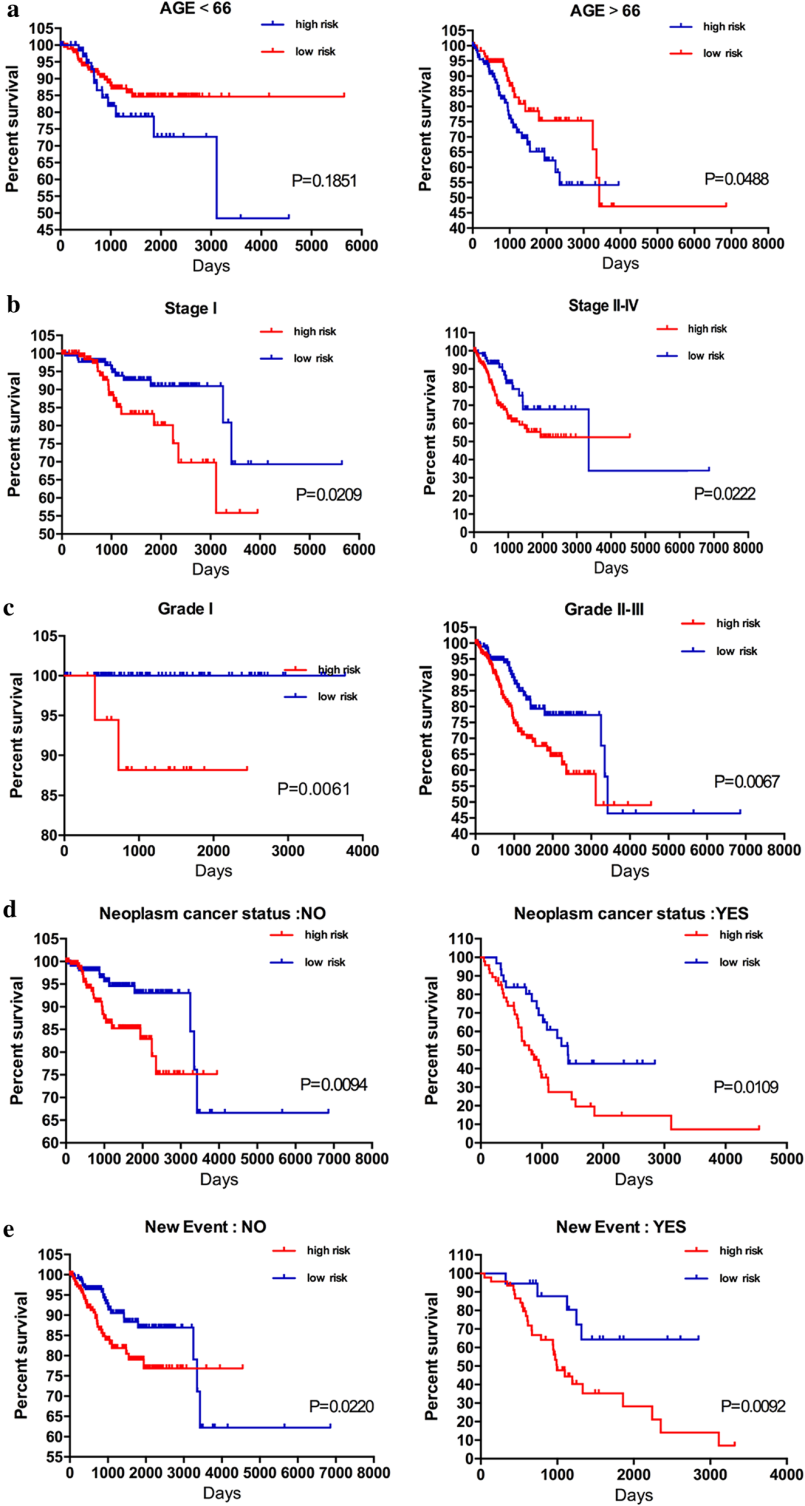
Kaplan–Meier curves for prognostic value of risk parameter signature for the patients divided by each clinical feature. **a** Age, **b** stage, **c** grade, **d** Person neoplasm cancer status, **e** new event

After further data mining and stratified analysis, the survival curves were not affected by stage (stage I or stage II–IV), and the nine-mRNA signature was validated as a reliable prognostic indicator for patients with EC. Patients in the high-risk group showed a poor prognosis (Fig. 6b). Similarly, despite the tumor grade, neoplasm cancer status, and tumor recurrence, the risk parameter based on the nine‐mRNA signature could be used to predict the prognosis of patients with EC (Fig. 6c–e). However, when we stratified patients with EC into two subgroups according to age (> 66 or ≤ 66 years), the risk parameter could no longer be independently used as an prognostic indicator for the subgroup of age < 66 years (Fig. 6a), indicating that the risk parameter is affected by the age of patients with EC; this point requires further exploration.

## Discussion

Recent studies showed that clinicopathological features such as age and metastatic diagnosis are not sufficient to precisely predict the outcome of patients with cancer. Thus, an increasing number of mRNAs have been identified as biomarkers of tumor progression or prognosis, and the clinical significance of the biomarkers has been evaluated [14]. For example, Nadaraja et al. [15] confirmed that low expression of ARAP1 is an independent prognostic biomarker of shorter progression-free survival in older patients with ovarian high-grade serous adenocarcinoma being administered first-line platinum-based antineoplastic therapy. Similarly, multivariate Cox proportional hazards regression model analysis was used to verified that patients with cervical cancer who had high tumor protein p73 expression had better outcomes, and thus this protein was considered as a prognostic indicator in patients with cervical cancer [16]. However, these biomarkers were still not sufficient for independently predicting patient prognosis. Particularly, single gene expression levels can be affected by multiple factors, preventing these markers from being used as reliable and independent prognosis indicators. Thus, a statistical model comprised of genetic markers for multiple related genes, combined with the predictive effect of each constituent gene, was used to improve prediction. The model is significantly more accurate than using single biomarkers in assessing the prognosis of patients with tumors [17, 18], leading to widespread use of the model.

The rapid development of high-throughput genetic sequencing technology has established a foundation for large biological data research [19]. Large amounts of genomic data were extracted from individual specimens to identify new diagnostic, prognostic, or pharmacological biomarkers [20]. In recent studies, a new prognostic signature was constructed by using microarray and RNA-sequencing data for gene expression levels or mutations. A Cox proportional hazards regression model was used for identification and verification [21, 22]. In the current study, we identified 10 functions showing significant differences in GSEA. As described above, rather than wide-range exploration, we selected the top‐ranking function to filter genes related to patient survival prediction. Univariate and multivariate Cox regression analyses were performed to determine the prognostic value of the combination of nine genes for patients with EC. This selected risk profile may be a more targeted and powerful prognostic assessment for predicting positive clinical outcomes and may be a more effective classification tool for patients with EC compared to other known prognostic assessment markers.

In this study, bioinformatics methods were used to explore the characteristics of mRNA risk factors and their clinical significance, and a new method for mining of potential prognostic markers was explored. This study y complements the previous understanding of EC and provides a foundation for future EC research. We used the EC dataset in TCGA to collect glycolysis-related genes and compare data from normal and EC tissues. Kaplan–Meier survival estimates revealed that patients with low-risk parameters had a better prognosis. The detection and calculation of risk parameter in EC patients have important clinical implications. However, because of the lack of patient metastasis and recurrence information in TCGA database, we could only use OS to assess patient prognosis, which is one limitation of our research. Additionally, in stratified analysis, the risk parameter could predict the prognosis of patients with EC in all subgroups except for the subgroup of age < 66 years. The reason for this difference is unclear requires further examination.

In addition, the nine-gene signature and same analysis method in liver cancer and colon cancer were used to obtain and verify the corresponding risk parameter (Additional file 3: Figure S2; Additional file 4: Figure S3). The results showed that the risk parameter based on the nine genes is not an independent prognostic indicator for liver cancer and colon cancer, confirming that the nine-gene signature is particularly important in EC.

Tumors are characterized by uncontrolled cell proliferation, which not only eliminates control of the cell cycle but also promotes cellular energy metabolism and finally leads to tumor cell growth and differentiation. Cellular energy is mainly derived from sugar metabolism, and most energy is supplied by ATP. In the 1920s, the German biologist Otto Warburg discovered abnormalities in energy metabolism in hepatoma cells. Although oxygen is present, tumor cells mainly rely on glycolysis for metabolism and consume large amounts of glucose accompanied by lactic acid production. This phenomenon of abnormal glucose metabolism was named as aerobic glycolysis or the Warburg effect [23]. Studies have shown that tumor cells can precisely regulate ATP synthesis by regulating substrate uptake and enzymes related to glycolysis, enabling them to adapt to the nutrient microenvironment, meet the energy and nutrient requirements for malignant proliferation, rapidly proliferate. Moreover, cancer metabolic reprogramming, which is closely associated with the Warburg effect, plays an important role in maintaining the interaction between oxygen-sensing transcription factors and the nutrient-sensing signal pathway [24]. This indicates that aerobic glycolysis uses a complicated mechanism of action. Tumor cell proliferation proceeds at a pace exceeding cellular energy supply, and thus excessive consumption of oxygen and nutrients by the cells can cause the tumor microenvironment to be hypoxic, low in sugar, and acidic, which is more pronounced in solid tumors [25]. Although not all tumors exhibit the Warburg effect, cellular energy abnormalities are widely recognized as one of the characteristics of tumor cells. After more than 90 years of continuous exploration and research, the Warburg effect has been found to occur in many malignancies, such as lung cancer, breast cancer, colon cancer, and gastric cancer. Recent studies showed that aerobic glycolysis plays an important role in EC occurrence and development. Metabolic profiling of EC cells revealed higher rates of glycolysis and lower glucose oxidation, and tumor cells may rely on GLUT6-mediated glucose transport and glycolytic–lipogenic metabolism for survival [26]. Highly differentiated EC showed significantly lower GLUT1 and GLUT3 expression than poorly differentiated tumors [27]. Several studies have predicted the survival of patients with EC using genes associated with cellular glycolysis. For example, high mobility group protein 1 suppression effectively inhibits the development and progression of EC [28]. The expression of lactate dehydrogenase 5 in EC is an independent prognostic indicator strongly associated with poor prognosis [29]. However, glycolysis-related gene markers for predicting EC prognosis have not been established. Using bioinformatics methods, we determined the genetic characteristics associated with cellular aerobic glycolysis (*CLDN9*, *B4GALT1*, *GMPPB*, *B4GALT4*, *AK4*, *CHST6*, *PC*, *GPC1*, and *SRD5A3*) and demonstrated their prognostic value in EC.

## Conclusion

We developed a nine-gene risk profile associated with cellular glycolysis which predicts the prognosis of patients with EC, with a higher risk parameter indicating poorer prognosis. The signature can be a used as a classification tool in clinical practice. These findings provide insight into the mechanisms of cellular glycolysis and identification of patients with poor prognosis in EC.

## Supplementary information

**Additional file 1: Table S1.** Primer sequence.

**Additional file 2: Figure S1.** Expression of nine genes in high and low risk groups (* represents for p < 0.01, ** represent for p < 0.001, **** represent for p < 0.00001).

**Additional file 3: Figure S2.** (a) The Kaplan–Meier curve for patients divided into high risk and low risk in liver cancer. 45(b) Different expression of nine selected genes in liver cancer.

**Additional file 4: Figure S3.** a) The Kaplan–Meier curve for patients divided into high risk and low risk in colon cancer. (b) Different expression of nine selected genes in colon cancer.

## Data Availability

The datasets generated and analysed during the current study are available in the TCGA (http://cancergenome.nih.gov/abouttcga) and cBioPortal (http://www.cbioportal.org).

## Abbreviations

EC: endometrial cancer
TCGA: The Cancer Genome Atlas
GSEA: gene set enrichment analysis
OS: overall survival
MSigDB: Molecular Signatures Database
FIGO: International Federation of Gynecology and Obstetrics
SIX1: oncofetal protein sine oculis-related homeobox 1
HER-2: human epidermal growth factor receptor-2
ARAP1: Ankyrin repeat and PH domain 1
GLUT1: glucose transporter type 1
GLUT3: glucose transporter type 3
