# Supplementary material for: Identification of novel cell glycolysis related gene signature predicting survival in patients with endometrial cancer
Source: Cancer Cell Int. 2019 Nov 14;19:296. doi: 10.1186/s12935-019-1001-0 (PMC6857303; doi:10.1186/s12935-019-1001-0)
Supplement: Supplementary file 1 — Additional file 1: Table S1. Primer sequence. [file 12935_2019_1001_MOESM1_ESM.docx]

**Table S1**

Primer sequence.

| **Gene Name** | **Primer Sequence** |
| --- | --- |
| **GAPDH** | F: GCACCGTCAAGGCTGAGAAC  R: TGGTGAAGACGCCAGTGGA |
| **CLDN9** | F: ATGGCTTCGACCGGCTTAG  R: CAGGGCATCTGGTCATCAGG |
| **B4GALT1** | F: CTAGCAACTTGACCTCGGT  R: CATTTGGGTTCTGCTTTGCC |
| **GMPPB** | F: TCAGTGGGTACGCATGGAGA  R: CCACATGAGAAGGCAGGTGT |
| **B4GALT4** | F: ACTTCGTGGGTGCCATTCAAGAGA  R: AAGGAGACACAGAAGGGCAGTTGT |
| **AK4** | F: CACTTCTTGCGGGAGAACATC  R: CCAACTCGGACATCATTAGGC |
| **CHST6** | F: ACATTCGTTCTACCTCGAGTCTCC  R: AGAGGTTCCTCAGCACCCCA |
| **PC** | F: GGCGACGGCGAGGAGATAG  R: GAGTAGATGGCTACGGTGCG |
| **GPC1** | F: GATGGCTGTCTGGATGACCT  R: GTAAGGGCCAGGAAGAGGAG |
| **SRD5A3** | F: TTTAATCAGGCCCTGTCTGC  R: GGGGTATAGAAATGGAATGGAGA |

F: Forward; R: Reverse.
