# Supplementary figures and images for: Identification of novel cell glycolysis related gene signature predicting survival in patients with endometrial cancer
Source: Cancer Cell Int. 2019 Nov 14;19:296. doi: 10.1186/s12935-019-1001-0 (PMC6857303; doi:10.1186/s12935-019-1001-0)

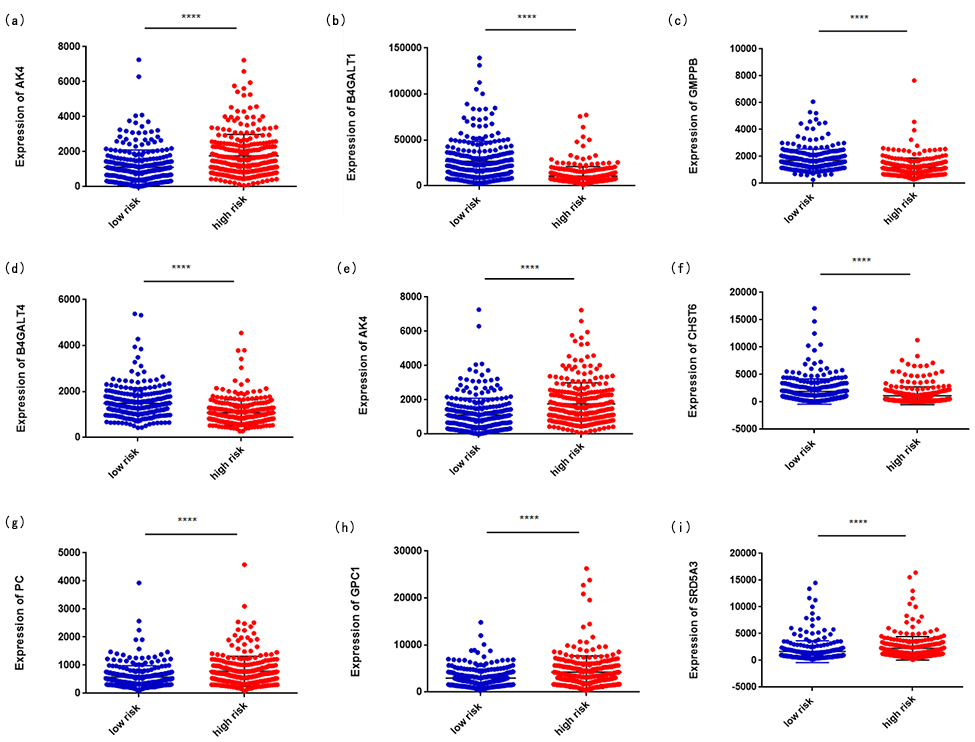

Supplement: Supplementary file 2 — Additional file 2: Figure S1. Expression of nine genes in high and low risk groups (* represents for p < 0.01, ** represent for p < 0.001, **** represent for p < 0.00001). [file 12935_2019_1001_MOESM2_ESM.jpg]

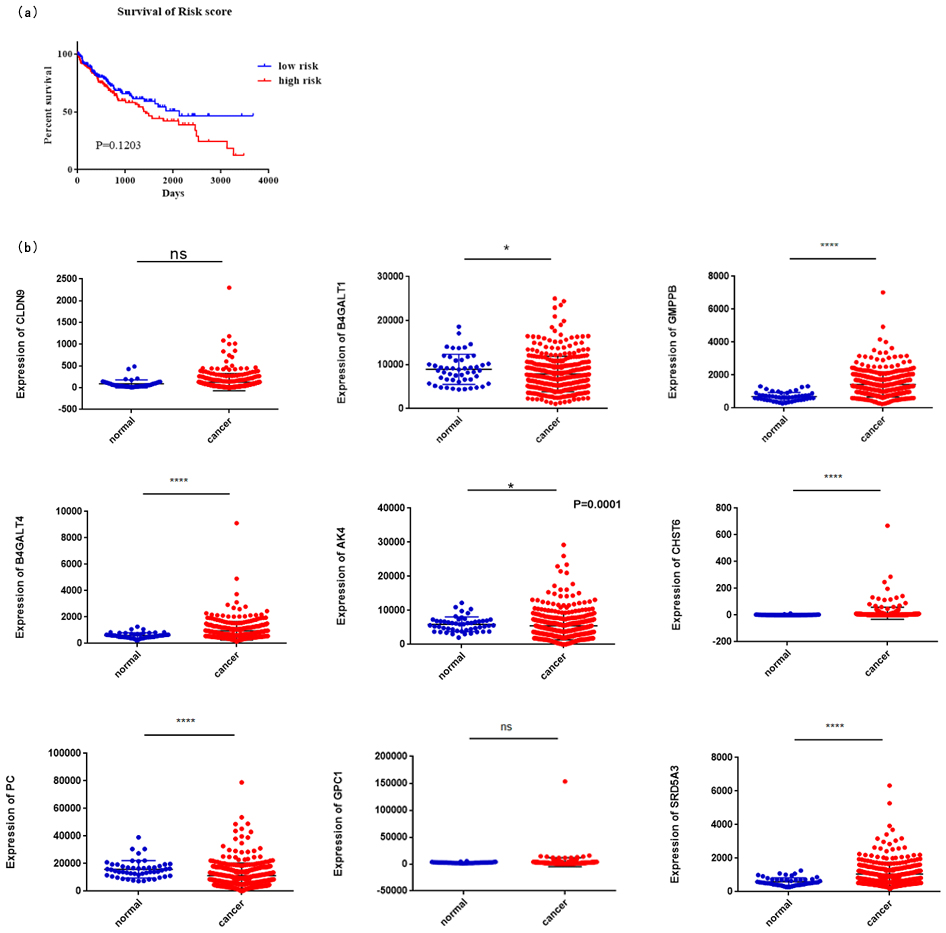

Supplement: Supplementary file 3 — Additional file 3: Figure S2. (a) The Kaplan–Meier curve for patients divided into high risk and low risk in liver cancer. 45(b) Different expression of nine selected genes in liver cancer. [file 12935_2019_1001_MOESM3_ESM.jpg]

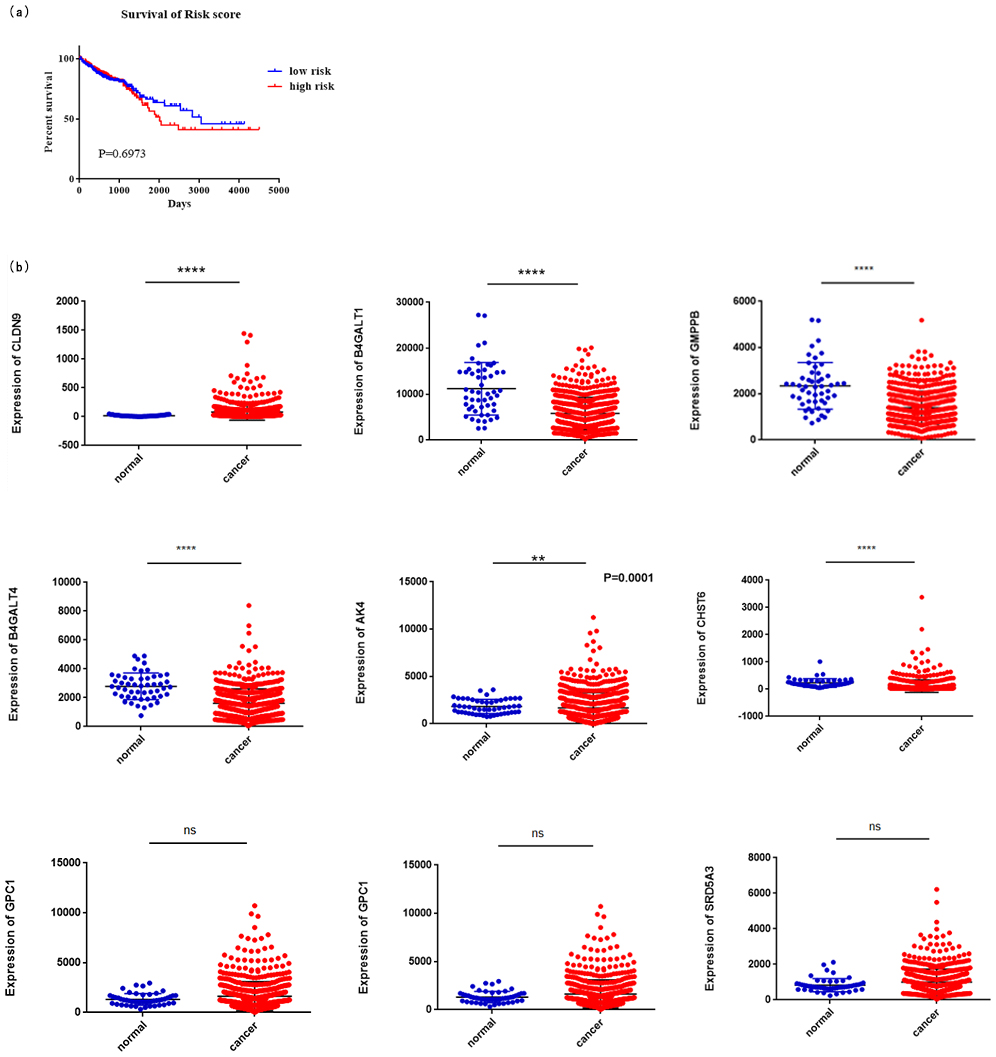

Supplement: Supplementary file 4 — Additional file 4: Figure S3. a) The Kaplan–Meier curve for patients divided into high risk and low risk in colon cancer. (b) Different expression of nine selected genes in colon cancer. [file 12935_2019_1001_MOESM4_ESM.jpg]
